# A cross-sectional analysis of registry data of severe mental disorders in Fuzhou, China: current status and prospects

**DOI:** 10.1186/s12888-022-04364-6

**Published:** 2022-12-14

**Authors:** Fuhao Zheng, Yawen Lin, Qinfei Wei, Zhaonan Zeng, Duanhua Xiong, Siying Wu

**Affiliations:** 1 Department of Epidemiology and Health Statistics, The Key Laboratory of Environment and Health, School of Public Health, Fujian Medical University, Fuzhou, 350001 China; 2 Department of Scientific Research Management, Fujian Provincial Hospital, Fujian Medical University, Fuzhou, 350001 China; 3 Center for Experimental Research in Clinical Medicine, Fujian Provincial Hospital, Fujian Medical University, Fuzhou, 350001 China; 4 Department of Prevention and Treatment, Fuzhou Neuropsychiatric Hospital, Fuzhou, 350001 China

**Keywords:** Severe mental disorders, Epidemiology, Disease management

## Abstract

**Objective:**

To investigate the proportion of registered cases relative to size, distribution characteristics, medication status, and management status of patients diagnosed with severe mental disorders (SMD) in Fuzhou. The medication status and management status were compared between patients in urban and non-urban areas to provide scientific evidence for improving SMD care, control, and treatment in primary health care institutions.

**Methods:**

Data (case types, demographic data, distribution data, medication status, and management status, etc.) of patients diagnosed with SMD in 12 districts, counties, and prefectures in the urban and non-urban areas of Fuzhou City were collected from October 2017 to September 2018. Three distributions (population, local, and districts/counties) were used to describe the proportion of registered cases relative to size and clinical characteristics of diagnosed SMD. Chi squared (*χ2)* test was used to compare the severity in urban and non-urban areas.

**Results:**

A total of 30,362 registered SMD patients were identified in Fuzhou City of which schizophrenia accounted for the highest number of cases (26,204, 86.31%), and paranoid psychosis had the least number of cases (47, 0.15%). Moreover, approximately half of SMD patients were 18 to 44 years old (45.38%). Close to one third of patients were farmers (30.23%), had a primary school or lower education level (54.17%), were poor, with most below the poverty line (55.35%). The proportion of diagnosed SMD relative to size was highest in Minqing County (0.53%) and lowest in Mawei District (0.38%). A total of 22,989 (75.72%) of the patients were taking medications, and only 17,509 (57.67%) were taking medications regularly. Moreover, the percentage of cases taking medications and those taking medications regularly were higher in urban areas than in non-urban areas (*P*<0.05). A total of 3065 patients were registered for management (10.09%). The managed proportion of SMD cases was higher in the urban areas than in the non-urban areas (*P* < 0.05).

**Conclusion:**

Schizophrenia is a key disease for comprehensive care and control of severe mental disorders in Fuzhou. The management of severe mental disorders should focus on poor groups with low educational backgrounds. Drug usage and management are better in urban areas than in non-urban areas, and thus management should be enhanced in non-urban areas. The medication management and case management of patients with severe mental disorders in Fuzhou need further improvements.

## Background

Severe mental disorders (SMD) refer to a group of mental illnesses with severely impaired social life, including schizophrenia, paranoid disorder, bipolar disorder, schizoaffective disorder, epilepsy and intellectual disability with mental disorders in China [1]. The fast-paced lifestyle has gradually increased the physical and psychological problems and stress levels of Chinese people due to the rapid progress of economic modernization and urban economy. Furthermore, the incidence of SMD have significantly increased [2]. The global burden of mental disorders was 7% in 2016, and SMD threatens more than 4% of adults worldwide. China has 5.81 million patients with SMD, and the overall average prevalence has increased [3] based on the Bureau of Disease Control and Prevention of the National Health Commission. The average mortality rate of SMD patients is nearly 2–3 times higher than that of the general population, indicating that the mortality rate pattern occurs in both high- and low-income patients [4]. SMD are common and are characterized by mood disorders and cognitive dysfunction. Most SMD patients require long-term treatment and nursing, which greatly affects the quality of life and productivity of the patients and their family members [5].

There are more new SMD cases each year, with a higher probability of recurrence and disability [6]. Drug therapy is a crucial treatment for SMD patients regardless of acute illness or long-term management [7]. Ensuring patients’ adherence to treatment is also essential for successful mental health management [8]. Several studies have shown that the chance of SMD recurrence is significantly reduced when patients take drugs for a long time [9].

However, drug non-compliance is common and can lead to serious clinical problems. Various studies have found that the compliance with the use of antipsychotic drugs is very low, as low as 20% [10]. Previous research has shown various factors, such as patient-, drug-, disease-, and environment-related, including age, educational status, drug quantity, drug side effects, psychotic symptoms, social support, and treatment combinations, affect the compliance of using related medications [11]. It can be seen that it is very important to implement targeted psychological care when SMD patients especially those with psychotic features are treated with psychotropic drugs. Psychological nursing of schizophrenia patients can stabilize their emotions, increase their emotional experience and expression, reduce their psychological distress, and then effectively improve their psychological state and relieve suffering from their illness. In addition, patients’ extreme behaviors, crimes, and accidents cause their caregivers to experience many negative emotions, such as anxiety and depression, which seriously affect their quality of life, physical and mental health, and social functioning. Therefore, these negative factors affect not only the individual but also the society.

The Chinese government has undertaken a series of actions over the past two decades to address disparities in access to and delivery of health care services for people with severe mental disorders. For example, the 2009 New Medical Reform Plan included major mental illnesses in public health care plans [4]. This shows that societal support is also very important.

With China’s economic development, the labour force from the mainland, rural and mountainous areas is gradually shifting to the economically developed areas along the southeast coast. The shift of the migrant population has led to an increase in the number of people living in the coastal areas and an increase in the number of people with mental illness. However, there are few studies on mental illness in the coastal areas of southeast China, and most of them focus on schizophrenia [12]. No research studies on the current situation of severe mental disorders in the coastal areas of southeast China have been found. Fuzhou is the capital of Fujian Province, located in the coastal region of southeastern China. Like most coastal cities in China, it has a large and representative migrant population. Therefore, we chose Fuzhou to investigate the situation of severe mental disorders, hoping to provide a basis for the prevention, control and management of severe mental disorders in Fujian Province, coastal cities in Southeast China and China.

This study assessed the proportion of registered cases relative to size, distribution characteristics, medication status, and management status of patients diagnosed with SMD in Fuzhou by comparing the medication status and management status in urban and non-urban areas.

## Methods

### Source of information

The number of cases, general conditions, medications, and management status of SMD patients in the counties (cities) and prefectures in Fuzhou City were obtained from October 2017 to September 2018 from National Severe Mental Disorders Information System (According to Chinese policy, once diagnosed, it must be registered in this national system). The data (clinical disease classification, demographic data, regional distribution, temporal distribution, treatment status, and management status of patients with severe mental disorders) were mainly from six urban areas in Fuzhou City (Gulou District, Jin’an District, Taijiang District, Cangshan District, Mawei District, and Changle District) and six non-urban counties at the prefecture level (Minhou County, Lianjiang County, Luoyuan County, Minqing County, Yongtai County, and Fuqing City). Urban and non-urban counties are differentiated according to Chinese household registration management. The population data of the counties (cities) and prefectures in Fuzhou were obtained from the Statistical Yearbook of the Fuzhou Bureau of Statistics.

### Participants

The inclusion criteria for the purpose of this study were: 1) Patients who were diagnosed by psychiatrist based on the 10th revised“International Statistical Classification of Diseases and Related Health Problems” (ICD-10) and included in the National Management of Severe Mental Disorders; 2) All registered patients who provided informed consent and focus was on home management. The exclusion criteria were as follows: patients who refused to register for management and those lost during follow-up for three consecutive years or were confirmed dead. Psychiatric staff at districts, counties, and prefectures in Fuzhou regularly conducted information verification and quality control management on the disease data of SMD patients to ensure reliability and reduce the occurrence of deviations.

### Measurement indicators

The measured indicators used for the purpose of the study were: 1) % of registered SMD cases defined as the number of new and old cases of SMD in a specific population at a certain time/total population at that time × 100%; 2) % receiving any medication defined as the number of SMD patients taking medication/total number of registered SMD patients × 100%. SMD patients taking medication are registered as taking medications, including regular medication and intermittent medication prescribed by mental health staff; 3) % receiving any medication on regular basis defined as the number of SMD patients regularly taking medication/total number of registered SMD patients × 100%). These will be referred to as regular medication patients i.e. patients who have been taking medication regularly this year. The physician of the community health service center where the patient lives regularly evaluate medication compliance, psychiatric condition and other conditions; 4) % managed cases (the number of managed registered cases of SMD/total number of SMD patients. SMD management refers to the detailed assessment of SMD patients. The psychiatrists comprehensively evaluated the management of patients with mental disorders in Fuzhou through tracking the proportion of patients with severe mental disorders such as risk, persistent lock-down, unstable condition, persistent non-medication, and intellectual disability (with mental disorders). The medical staff of the community health service center regularly assessed medication compliance and injury or injury-related behaviors of SMD patients.

### Statistical analysis

Excel 2019 software was used to organize and summarize data. ArcMap10.2 software was used to draw grade maps. SPSS20.0 software was used for data analysis, calculating the proportion of registered SMD cases and composition ratio (It is defined as a ratio involving two or more levels of each sociodemographic variable e.g. schizophrenia, intellectual disability (with mental disorders), bipolar disorder, mental disorders caused by epilepsy, schizoaffective psychosis and paranoid psychosis composition ratio), and descriptive analysis of regional and patient demographic characteristics. Chi squared (*χ2)* test was used to compare proportions. The test level was two-sided at α = 0.05.

## Results

### Distribution characteristics of SMD types in Fuzhou

A total of 30,362 registered SMD patients were identified in Fuzhou City. The proportion of registered cases relative to the population size was 0.42%. Schizophrenia accounted for the highest number (26,204, 86.31%). The number of cases, proportions and composition of other diseases are shown in Table 1.

**Table 1:** Proportion of Severe Mental Disorders in Fuzhou City

Diseases types	Number of Cases (person)	Percentage of Registered Cases Relative to the Population Size (%)	Composition ratio(%)
Schizophrenia	26,204	0.36	86.31
Intellectual disability (with mental disorders)	2121	0.03	6.98
Bipolar disorder	1292	0.02	4.26
Mental disorders caused by epilepsy	530	0.01	1.75
Schizoaffective psychosis	168	0.00	0.55
Paranoid psychosis	47	0.00	0.15
Total	30,362	0.42	100.00

### The three distributions of SMD in Fuzhou

#### Population distribution

A total of 30,362 registered SMD patients were identified in Fuzhou. The specific sociodemographic distribution is shown in Table 2.

**Table 2:** Demographic data of patients with severe mental disorders in Fuzhou

Variables	Number of Cases (persons)	Composition ratio(%)
Gender		
Male	16,633	54.78
Female	13,729	45.22
Age		
Under 18	377	1.24
8 (inclusive)-44 years old	13,778	45.38
45–59 years old	10,248	33.75
60–64 years old	2456	8.09
65 years old and above	3503	11.54
Education level		
Elementary school and below	14,593	48.06
Junior high school or technical secondary school	12,841	42.29
University and above	646	2.13
Unknown	1855	6.11
Marital status		
Unmarried	11,582	38.15
Married	15,155	49.91
Widowed	746	2.46
Divorce	1531	5.04
Unspecified marital status	1348	4.44
Family history of severe mental disorders		
YES	1434	4.71
NO	27,058	89.12
Unknown	1873	6.17
Economic status		
Poor	16,796	55.32
Non-poverty	12,792	42.13
Unknown	774	2.55
Profession		
On-duty workers	588	1.94
On-the-job manager	61	0.20
Farmer	9177	30.23
Laid-off or unemployed	7644	25.18
School student	177	0.58
Retirement	636	2.09
Professional skill worker	285	0.94
Other Unknown area	7274	23.96
Unknown	4520	14.89
Area		
Urban area	15,949	52.53
Non-urban	14,413	47.47

#### Local distribution

**Fig. 1:**
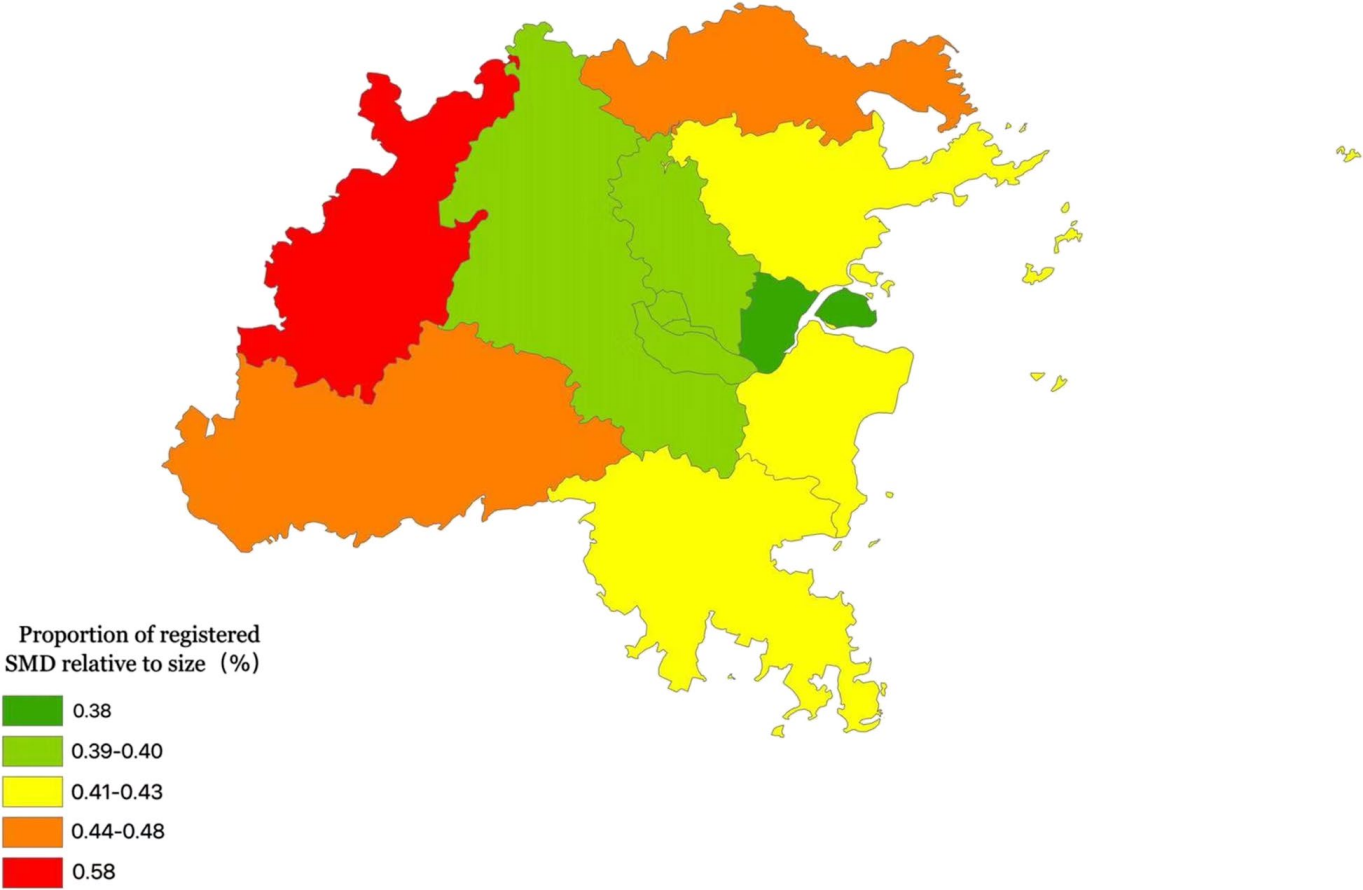
Grade map of the number of patients with severe mental disorders in each district and county of Fuzhou

As of September 2018, among the 30,362 registered patients with severe mental disorders in Fuzhou, there were 15,949 (0.41%) in 6 urban areas and 14,413 (0.43%) in 6 non-urban areas. The proportion of diagnosed SMD in urban areas was lower than that in non-urban areas. Minqing County had the highest proportion relative to its population size, followed by Yongtai County, while Mawei District had the lowest proportion of registered cases (Table 3 and Fig. 1). Among the different types of severe mental disorders, the proportion of registered cases with schizophrenia was the highest in the districts and counties of Fuzhou. The specific distribution is shown in Table 4.

**Table 3:** The proportion of registered severe mental disorders (SMD) in various districts and counties in Fuzhou

Region	Permanent population (10,000 people)	Number of Cases (person)	Proportion of Registered SMD Relative to Size (%)
Gulou District	74.00	2974	0.40
Taijiang District	48.60	1947	0.40
Cangshan District	83.60	3379	0.40
Mawei District	26.20	996	0.38
Jin’an District	87.70	3457	0.39
Changle District	73.90	3196	0.43
Minhou County	72.50	2847	0.39
Lianjiang County	59.30	2462	0.42
Luoyuan County	21.20	1008	0.48
Minqing County	24.00	1389	0.58
Yongtai County	25.40	1226	0.48
Fuqing City	131.60	5481	0.42

**Table 4:** Proportion of Registered Severe Mental disorders (SMD) by Disorder Type in all districts and counties of Fuzhou

Region	Schizophrenia		Intellectual disability (with mental disorders)		Bbipolar disorder		Mental disorders caused by epilepsy		Schizoaffective psychosis		Paranoid psychosis	
	Number of Cases	Proportion of Registered SMD Relative to Size %	Number of Cases	Proportion of Registered SMD Relative to Size %	Number of Cases	Proportion of Registered SMD Relative to Size %	Number of Cases	Proportion of Registered SMD Relative to Size %	Number of Cases	Proportion of Registered SMD Relative to Size %	Number of Cases	Proportion of Registered SMD Relative to Size %
Fuqing City	26,204	86.31	2121	6.99	1292	4.26	530	1.75	168	0.55	47	0.15
Gulou District	2501	84.10	225	7.57	152	5.11	76	2.56	16	0.54	4	0.13
Taijiang District	1393	71.55	476	24.45	44	2.26	30	1.54	2	0.10	2	0.10
Cangshan District	2913	86.21	220	6.51	190	5.62	38	1.12	17	0.50	1	0.03
Mawei District	824	82.73	121	12.15	32	3.21	15	1.51	4	0.40	0	0.00
Jin’an District	2776	80.30	448	12.96	154	4.45	55	1.59	20	0.58	4	0.12
Changle District	2426	85.21	129	4.53	141	4.95	116	4.07	21	0.74	14	0.49
Minhou County	2293	93.14	30	1.22	88	3.57	29	1.18	15	0.61	7	0.28
Lianjiang County	954	94.64	6	0.60	25	2.48	16	1.59	6	0.60	1	0.10
Luoyuan County	1187	85.46	3	0.22	160	11.52	13	0.94	23	1.66	3	0.22
Minqing County	1150	93.80	20	1.63	32	2.61	16	1.31	8	0.65	0	0.00
Yongtai County	4771	87.05	412	7.52	179	3.27	90	1.64	24	0.44	5	0.09
Fuqing City	3016	94.37	31	0.97	95	2.97	36	1.13	12	0.38	6	0.19

#### Temporal distribution

**Fig. 2:**
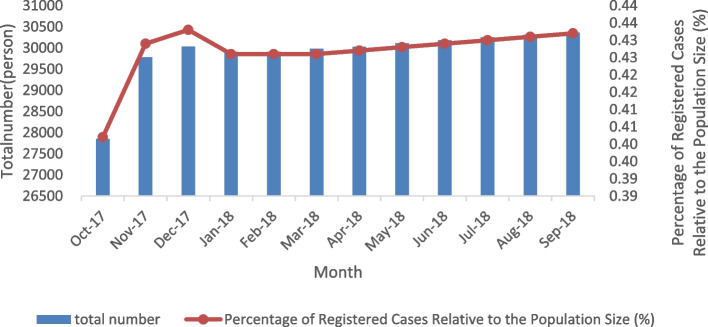
Monthly proportion of registered cases with severe mental disorders from October 2017 to September 2018 in Fuzhou

From the data obtained from October 2017 to September 2018, it can be seen that proportion of diagnosed SMD was increasing month-by-month (Fig. 2 and Table 5).

**Table 5:** Proportion of Severe Mental Disorders in Fuzhou City by Month

Month	Total number (person)	Percentage of Registered Cases Relative to the Population Size (%)
October 2017	27,847	0.40
November 2017	29,780	0.43
December 2017	30,032	0.43
January 2018	29,910	0.43
February 2018	29,908	0.43
March 2018	29,982	0.43
April 2018	30,029	0.43
May 2018	30,111	0.43
June 2018	30,177	0.43
July 2018	30,250	0.43
August 2018	30,311	0.43
September 2018	30,362	0.43

### Medications for SMD patients in Fuzhou

A total of 22,989 of 30,362 SMD patients were taking medication (75.72%). A total of 17,509 were taking the medication regularly (57.67%). As shown in Tables 6 and 7 the percentage of cases taking medication and those taking medications o regularly was significantly higher in urban areas than in non-urban areas (*P* < 0.05), respectively.

**Table 6:** Drugs taken by patients with severe mental disorders in Fuzhou

Area	Number of registered patients (persons)	Number of medicated cases (persons)	Medicated cases (%)	*χ*^*2*^	*P*
Urban area	15,949	12,579	78.87	181.75	<0.001
Non-urban	14,413	10,410	72.23

**Table 7:** Regular medication status of patients with severe mental disorders in Fuzhou

Area	Number of registered patients (persons)	Number of regularly medicated cases (person)	Percentage of regularly medicated cases (%)	*χ*^*2*^	*P*
Urban area	15,949	10,486	65.75	898.43	<0.001
Non-urban	14,413	7023	48.73		

All SMD types were found in urban areas. Medication and regular-takings were highest among patients with mental disorders caused by epilepsy (91.60 and 80.40%, respectively) with statistically significant differences (*P* < 0.05) (Tables 8 and 9).

**Table 8:** Medications taken by patients with various types of severe mental disorders in Fuzhou

Classification	Number of registered patients (persons)	Number of medicated cases (persons)	Percentage of medicated cases (%)	*χ*^*2*^	*P*
Schizophrenia				105.52	<0.001
Urban area	13,423	10,683	79.59		
Non-urban	12,781	9489	74.24		
Paranoid psychosis				4.82	<0.05
Urban area	17	13	76.47		
Non-urban	30	13	43.33		
Schizoaffective psychosis				4.63	<0.05
Urban area	71	55	77.46		
Non-urban	97	60	79.38		
Bipolar disorder				16.00	<0.001
Urban area	667	507	76.01		
Non-urban	625	412	65.92		
Mental disorders caused by epilepsy				37.79	<0.001
Urban area	250	229	91.6		
Non-urban	280	197	70.36		
Intellectual disability (with mental disorders)				242.52	<0.001
Urban area	1521	1093	71.86		
Non-urban	600	212	35.33

**Table 9:** Regular medications of patients with various types of severe mental disorders in Fuzhou

Classification	Number of registered patients (persons)	Number of regularly medicated cases (persons)	Percentage of regularly medicated cases (%)	*χ*^*2*^	*P*
Schizophrenia				718.62	<0.001
Urban area	13,423	9020	67.2		
Non-urban	12,781	6508	50.92		
Paranoid psychosis				4.32	<0.05
Urban area	17	11	64.71		
Non-urban	30	10	33.33		
Schizoaffective psychosis				6.12	<0.05
Urban area	71	48	67.61		
Non-urban	97	47	48.45		
Bipolar disorder				60.45	<0.001
Urban area	667	434	65.07		
Non-urban	625	272	43.52		
Mental disorders caused by epilepsy				73.94	<0.001
Urban area	250	201	80.4		
Non-urban	280	123	43.93		
Intellectual disability (with mental disorders)				292.12	<0.001
Urban area	1521	772	50.66		
Non-urban	600	63	10.5		

### Management of SMD patients in Fuzhou

A total of 3065 of 30,362 SMD patients were registered for management (10.09%) (2244 (16.37%) in urban areas and 821 in non-urban areas (6.04%). As shown in Table 10, the percentage of managed registered SMD cases was significantly higher in urban areas than in non-urban areas (*P* < 0.05).

**Table 10:** Management of patients with severe mental disorders in Fuzhou

Area	Number of managed cases	Number of non-managed cases	Percentage of managed cases (%)	*χ*^*2*^	*P*
Urban area	2244	13,705	16.37	584.92	<0.001
Non-urban	821	13,592	6.04		

## Discussion

As an open coastal city, the number of people on the move in Fuzhou is increasing year by year and the number of people with serious mental disorders is attracting attention. The rise in the number of people with severe mental disorders has also had a number of negative social and economic effects. In this study, a total of 30,362 registered SMD patients were identified in Fuzhou City, of which schizophrenia accounted for the highest number of cases (26,204, 86.31%). Most SMD patients in Fuzhou have a low educational level, are poor, and are married. The proportion of diagnosed SMD relative to size was highest in Minqing County (0.53%) and lowest in Mawei District (0.38%). The percentage of cases taking medications and those taking medications regularly and the managed proportion of SMD cases in Fuzhou are higher in urban areas than in non-urban areas. (*P*<0.05).

### Schizophrenia requires comprehensive prevention and control in Fuzhou

Herein, of 30,362 registered SMD patients, schizophrenia had the highest proportion of cases (86.31%), followed by intellectual disability (with mental disorders) (6.98%), while paranoid psychosis had the least (0.15%), consistent with research results of many scholars, such as Liao Zhixian [13]. Among them, the relatively low proportion of patients with bipolar disorder may be due to reasons such as loss to follow-up. A detailed analysis on site visits will be conducted in a follow-up analysis. Therefore, severe schizophrenia and intellectual disability (associated with mental disorders) are key mental disorders in Fuzhou that need comprehensive screening, tracking, and control.

Schizophrenia is a chronic mental illness with the most severe symptoms and is the most difficult to treat. Severe schizophrenia may lead to suicide or other injuries [14]. Strong family social institutions and functions, timely treatment, systematic, and high compliance can positively enhance treatment outcomes [15].

Presently, typical or atypical antipsychotics are mainly used to treat SMD [16]. Meanwhile, targeted psychological care can effectively stabilize the patient’s mood, increase emotional experience and expression, reduce psychological pressure, and effectively improve the patient’s psychological state and reduce the disease burden [17]. Many experts and scholars, including Laura Asher [18], have shown that support from family and other communities can effectively reduce the severity of schizophrenia symptoms in various patients. Therefore, the patient’s family and community should improve their relationship with the patient to enhance monitoring and management of the symptoms of SMD patients.

Most SMD cases can be treated and relieved with peer support due to the continuous development and technological advancement of modern therapeutics in China in recent years [19].

### Comprehensive prevention and control of SMD in Fuzhou should focus on poor and patients with low educational backgrounds

This study showed that more men suffer from SMD than women in Fuzhou, suggesting that it may be that women with SMD may not be easily detected due to less pronounced aggressive features [20].

Approximately, half of SMD patients were in the age range of 18 to 44 years old (45.38%), while approximately a third were 45 to 59 years old (33.75%). These two age groups tend to experience important moments in their lives, and bear more stress from school, family, work, and life, and face more emergencies, leading to a heavy stress load. Therefore, the Fuzhou Municipal Government should strengthen mental health education among young people and adults and address family mental health problems.

More than half SMD patients had a primary education level and below (including semi-illiterate) (54.17%). Most people with low educational levels have little knowledge of mental health. Therefore, they would not seek medical services when they mental health problems occur. In contrast, people with a higher level of education know more about mental illness and can seek medical attention as soon as symptoms manifest. The number of SMD patients is relatively small among people with a high level of education compared with those with low educational level.

Also, close to one third of SMD patients were farmers (30.23%) and laid-off or unemployed (25.18%). The proportion of the poor and those below the local poverty line was 55.35%, higher than other cases.

In summary, most SMD patients had low educational level or were poor. Therefore, the government should pay more attention to these disadvantaged groups, improve the medical insurance system, and raise mental health awareness to reduce the incidence of mental illness. Besides, the relatively high rates of unmarried and divorced patients with SMD indicate that society discriminates against them and is unwilling to interact with them. The government and communities should also spread mental health awareness among the general public to eliminate discrimination.

### The proportion of diagnosed SMD in Fuzhou was lower in urban areas than in non-urban areas

Herein, Minqing County had the highest proportion of diagnosed SMD relative to population size (0.58%), while Mawei District had the lowest (0.38%). The proportion of diagnosed SMD cases (0.41%) was lower in the six urban areas than in the six non-urban areas (0.43%). Therefore, SMD was less common in urban areas because of higher sociocultural and economic conditions that may have enabled people in urban areas to seek early detection, early diagnosis, and early treatment, preventing deterioration to SMD status. Therefore, a joint mental health conference system is necessary for the districts, counties and sub-districts of Fuzhou.

Furthermore, the proportion of SMD registered cases in Fuzhou City increased from October 2017 to September 2018 with apparent month-by-month upward rise in registered cases of SMD. This may be due the longer course of most types of SMD. It is also possible that these changes over time may have been driven by service characteristics [21]. SMD often recurs, more difficult to cure, and contributes to lower migration among this patient’s population [22]. Therefore, community and hospital staff should conduct regular-follow up, assess the medication status of SMD patients discharged from the hospital, and educate family members on the importance of ensuring the patients’ medication compliance. Meanwhile, public awareness of SMD should be further strengthened to prevent and reduce SMD cases.

### The medication and management status of SMD patients in Fuzhou are higher in urban areas than in non-urban areas

World Health Organization (WHO) defines medication compliance as the degree of consistency between an individual’s medication use and the doctor’s prescription [23]. Most scholars have focused on the medication compliance of SMD patients as early as the 1970s. Medication treatment is the most effective method for improving SMD. Besides, the Chinese government has issued corresponding subsidy policies, to reduce the cost of treating SMD patients, burden, preventing patients’ irregular visits to doctors and random medication, reducing disease fluctuations, attracting patients into community management [24, 25].

Herein, the percentages of registered SMD cases taking medications and those taking medications on regular basis were 75.72 and 57.67%, respectively. Presently, the corresponding national figures are 81.30 and 41.78%, respectively. Furthermore, the proportion of medicated and regularly medicated SMD case s were significantly different between urban and non-urban areas in Fuzhou. Based on different SMD types, the medication and regular medication proportions were significantly higher in the six urban areas than in the non-urban area. This could be due to patients in urban areas having better medical resources than non-urban areas, guaranteeing better quality of medical treatment [26]. Moreover, community rehabilitation staff in urban areas are well-trained and can supervise SMD patients better than in non-urban areas [27]. Therefore, overall, patients in urban areas have access to more standardized management than in non-urban settings in China. Also, patients in urban areas tend to have higher education levels, may be better equipped to obtain disease-related knowledge through multiple channels, and have higher medication compliance than patients in non-urban areas [28].

Herein, the percentage of medicated and regularly medicated SMD cases was highest for mental disorders caused by epilepsy, consistent with the results of Nesvåg Ragnar and other scholars [16], suggesting that these cases will take the drug actively because of the fear of epileptic seizure experience. Some patients have high level of internalized stigma and have poor medication compliance [29]. The more serious the positive symptoms before treatment, the worse the medication compliance [30].

In most people with SMD, it is difficult to implement an effective recovery program without antipsychotic medication [31]. The rate of patients with recurrent episodes or severe deterioration may be higher in the future, and antipsychotic drugs can prevent its recurrence [32]. Population and sociological factors, disease-related factors, psychological factors, drug-related factors, and other social support affect medication compliance in schizophrenia patients [33]. Therefore, medical staff should assess the medication compliance of patients with mental disorders and the main influencing factors of non-compliance and offer individualized intervention strategies [34].

The management status of SMD patients in Fuzhou was significantly lower (10.09%) than the current national management status (82.69%). The possible reason is related to the lack of a clear and orderly management mechanism for SMD in the local area. Also, the percentage of managed cases with SMD was significantly higher in urban areas than in non-urban areas, possibly because the management system is more effective in urban areas than in non-urban areas.

Moreover, patients in non-urban areas often go out for work, making it difficult for community rehabilitation workers and medical staff to manage them in a unified manner. Therefore, the government should strengthen the work quality of non-urban psychiatrists and improve the follow-up work in the community, targeted training of medical staff and family care management [11] to enhance the management rate of registered patients.

## Strengths and limitations

Strengths of this study include its reliance on data from the medical digital records, which were sourced from the National Severe Mental Disorders Information System ensuring accuracy and reliability of the presented data. The research we conducted was based on large database of SMDs in Fuzhou, one of the largest southeastern coastal cities of China, with good representation of diagnosed SMD cases, which is also a strength of this study.

However, our results were based on cross-sectional analysis of available data and could not rule out reverse causality and bidirectional associations. Furthermore, our system does not include information about psychosocial interventions received by registered SMD cases or any information about co-morbid substance use. Therefore, limiting our ability to relate our findings to the availability of comprehensive services to these cases and to make policy recommendations for improving and better planning of mental health services in Fuzhou, China.

## Conclusion

Schizophrenia accounts for the highest proportion of all SMD in Fuzhou and requires comprehensive prevention. Intellectual disability (may be accompanied by mental disorders) also accounts for a high proportion of SMD. Therefore, the government should focus on Schizophrenia and Intellectual disability, provide support to families, and formulate targeted treatment programs. However, other SMD types should not be completely ignored. Most SMD patients in Fuzhou have a low educational level, are poor, and are married. Therefore, the government should focus on these disadvantaged groups and strengthen treatment. The government should also improve the medical insurance system, establish a community rehabilitation system, and create a suitable community environment for patient rehabilitation. The percentage of cases taking medications and those taking medications regularly and the managed proportion of SMD cases in Fuzhou are higher in urban areas than in non-urban areas. However, the rates are lower than those of the national level. Therefore, the government should enhance mental health prevention and control work in Fuzhou, improve health institutions to reduce the proportion of diagnoses SMD, and thus protecting people’s health.

## Data Availability

The datasets generated and/or analysed during the current study are not publicly available due to individual privacy but are available in summary/group level form from the corresponding author on reasonable request.
